# Supplementary material for: Modeling sensory-motor decisions in natural behavior
Source: PLoS Comput Biol. 2018 Oct 25;14(10):e1006518. doi: 10.1371/journal.pcbi.1006518 (PMC6219815; doi:10.1371/journal.pcbi.1006518)
Supplement: S1 Appendix — (PDF) [file pcbi.1006518.s001.pdf]

Modeling sensory-motor decisions in natural behavior

Supporting Information

Appendix 1: Simulation Results

Using a canonical 2D gridworld in reinforcement learning (RL) research, the goals are to empirically prove that modular IRL algorithm can estimate rewards and discount factors correctly, demonstrate its advantages over standard IRL, and show an example of sparse modular IRL. Part of the gridworld is shown in Fig 1. Different module objects are indicated by different colors and shapes. Behavioral data (state-action pair samples) are collected from a modular RL agent.

We first show that modular IRL is able to recover module rewards and discount factors correctly. The environment contains six modules each with ten objects. Three of them have positive rewards and the other three have negative rewards. 10 gridworlds are generated with random layouts of objects. The agent navigates each world for 6,000 steps. Non-sparse modular IRL (Eq (9)) is used to estimate $r^{(1:6)}$ and $\gamma^{(1:6)}$ and we calculate the mean estimation and standard error. The results are shown in Table 1, it is evident that modular IRL is highly accurate in recovering the true rewards and discount factors given a large amount of data.

Modular vs. Bayesian inverse reinforcement learning In modeling natural human behaviors, one particularly important aspect of a machine learning algorithm is its sample efficiency, given that it could be expensive to collect behavior data unlike in computer simulation. The performance of modular IRL on sample efficiency is compared with a standard non-modular Bayesian IRL [1]. We use a Laplacian prior in Bayesian IRL since the rewards are sparse. Fig 2 shows the results. The test environment has 4 modules and each has 4 objects which is made smaller because Bayesian IRL is computationally expensive. Both algorithms are given different amount of samples (state-action pairs) for training. Then policies generated using the learned rewards are compared. Policy agreement is defined as the proportion of the states that have the same policy as the ground truth, which is used because the outputs of these two algorithms are weights and rewards that can not be directly compared. Modular

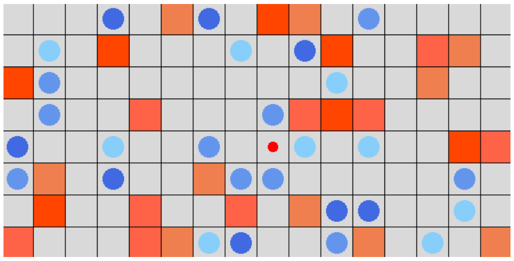

Fig 1. Part of the 2D gridworld test domain. Red squares are obstacles with negative reward. Blue circles are targets with positive reward. The small red dot is the modular RL agent. Different colors indicate different modules with distinct rewards and discount factors. The objects of the same module have the same color.

Table 1. Estimated rewards and discount factors comparing to the ground truth for the six modules in the 2D gridworld experiment. The results are presented as mean ± standard error ($N = 10$). The estimations are highly accurate due to the availability of a large amount of data.

	$r^{(1)}$	$r^{(2)}$	$r^{(3)}$
Truth	+5	+10	+15
Estimation	+5.00±0.02	+9.94±0.03	+15.02±0.03
	$r^{(4)}$	$r^{(5)}$	$r^{(6)}$
Truth	-5	-10	-15
Estimation	-4.97±0.02	-10.03±0.03	-14.85±0.07
	$\gamma^{(1)}$	$\gamma^{(2)}$	$\gamma^{(3)}$
Truth	0.7	0.6	0.5
Estimation	0.70±0.00	0.60±0.00	0.50±0.00
	$\gamma^{(4)}$	$\gamma^{(5)}$	$\gamma^{(6)}$
Truth	0.3	0.2	0.1
Estimation	0.30±0.00	0.20±0.00	0.10±0.00

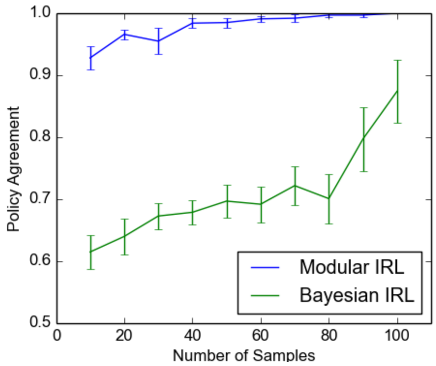

Fig 2. Modular IRL vs Bayesian IRL on sample efficiency, measured by average policy agreement ± standard error ($N = 10$). Modular IRL has significant higher sample efficiency.

IRL obtained nearly 100% policy agreement with far fewer data samples compared to the Bayesian IRL.

Sparse modular inverse reinforcement learning Next we evaluate the performance of sparse modular IRL algorithm (Eq (10)) in terms of sample efficiency. Again the gridworld contains 10 modules and each has 10 objects. The agent only considers 2 modules, i.e., the agent makes decision by treating all other modules to have zero rewards. Therefore, the hypothetical module set has size $|\mathcal{H}| = 10$ and actual module set has $|\mathcal{H}'| = 2$.

The mean squared error (MSE) of the estimated reward is shown in Fig 3. If data is scarce, the sparse version of modular IRL algorithm ($\lambda = 0.1, 0.25$) can recover rewards more accurately than the non-sparse version. Sparse modular IRL correctly identifies modules that the agent paid attention to, indicated by low MSE values obtained. As the regularization constant λ controls the importance of the regularization term, a very large λ introduces a large bias in estimation and may fail to converge to the truth, as shown by $\lambda = 1$. One can use the standard cross-validation techniques in choosing the value for λ.

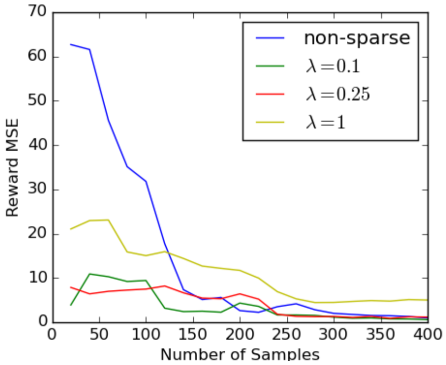

Fig 3. Modular IRL vs sparse modular IRL on sample efficiency, measured by mean squired error (MSE) of estimated reward. Sparsity can greatly improve sample efficiency with a carefully chosen value of λ.

References

1. Ramachandran D, Amir E. Bayesian inverse reinforcement learning. In: Proceedings of the 20th International Joint Conference on Artificial Intelligence. Morgan Kaufmann Publishers Inc.; 2007. p. 2586–2591.
