# Supplementary material for: Modeling sensory-motor decisions in natural behavior
Source: PLoS Comput Biol. 2018 Oct 25;14(10):e1006518. doi: 10.1371/journal.pcbi.1006518 (PMC6219815; doi:10.1371/journal.pcbi.1006518)
Supplement: S2 Appendix — (PDF) [file pcbi.1006518.s002.pdf]

Modeling sensory-motor decisions in natural behavior

## Supporting Information

## Appendix 2: One-way ANOVA for Estimated Rewards

Table 1 shows ANOVA results for individual differences in reward between subjects. Fig 1 visualizes the effect of task condition on reward function for each individual subject.

**Table 1.** One-way ANOVA for individual differences in reward between subjects and across task instructions. Between-subject differences for all modules are significant in all task conditions.

| | Target $r$ | Obstacle $r$ | Path $r$ |
|---|---|---|---|
| Task 1 | $F(25,4) = 6.53$ | $F(25,4) = 5.60$ | $F(25,4) = 4.57$ |
| | $p = 3.38 \times 10^{-11}$ | $p = 1.16 \times 10^{-9}$ | $p = 8.44 \times 10^{-8}$ |
| Task 2 | $F(25,4) = 8.09$ | $F(25,4) = 12.11$ | $F(25,4) = 12.12$ |
| | $p = 1.41 \times 10^{-13}$ | $p = 1.18 \times 10^{-18}$ | $p = 1.16 \times 10^{-18}$ |
| Task 3 | $F(25,4) = 7.65$ | $F(25,4) = 5.91$ | $F(25,4) = 3.17$ |
| | $p = 6.11 \times 10^{-13}$ | $p = 3.50 \times 10^{-10}$ | $p = 4.50 \times 10^{-5}$ |
| Task 4 | $F(25,4) = 21.38$ | $F(25,4) = 5.03$ | $F(25,4) = 7.20$ |
| | $p = 6.57 \times 10^{-27}$ | $p = 1.21 \times 10^{-8}$ | $p = 3.00 \times 10^{-12}$ |

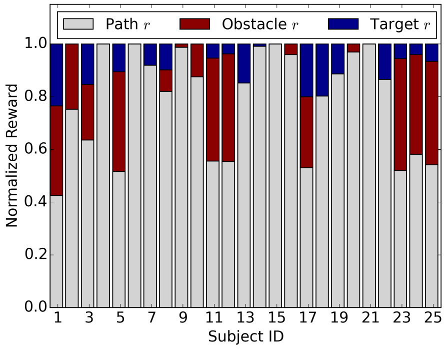


**(A)** Task1: path only

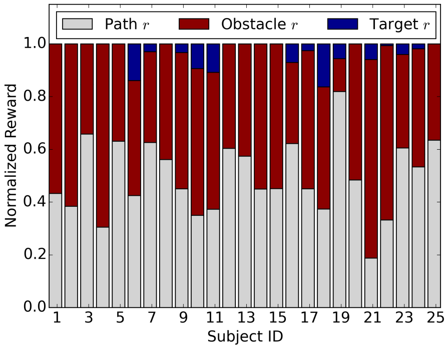


**(B)** Task2: obstacle + path

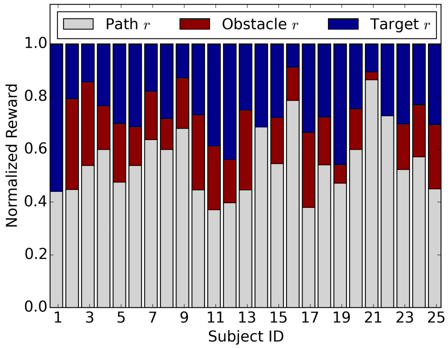


**(C)** Task3: target + path

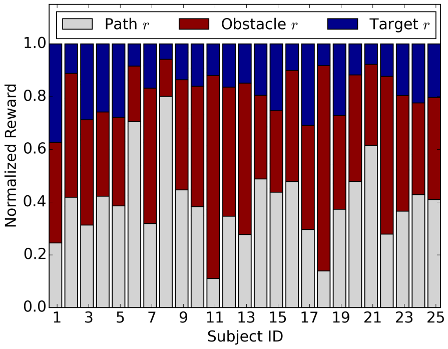


**(D)** Task4: target + obstacle + path

**Fig 1.** Average normalized rewards for each subject under different task instructions. The relative reward magnitude changes between tasks and agrees with task instructions. Under the same task instruction, individual differences in reward function are shown.
