# Supplementary material for: Modeling sensory-motor decisions in natural behavior
Source: PLoS Comput Biol. 2018 Oct 25;14(10):e1006518. doi: 10.1371/journal.pcbi.1006518 (PMC6219815; doi:10.1371/journal.pcbi.1006518)
Supplement: S3 Appendix — (PDF) [file pcbi.1006518.s003.pdf]

Modeling sensory-motor decisions in natural behavior

Supporting Information

Appendix 3: Bayesian Inverse Reinforcement Learning

Bayesian IRL leverages demonstrated state-action pairs, treats them individually as evidence for the underlying reward function and therefore is able to express the likelihood of reward functions given demonstrations. The normalizing factor for computing the probability of reward functions is hard to compute, hence Bayesian IRL instead adopts a Monte Carlo Markov Chain (MCMC) sampling method to acquire a set of reward samples using the unnormalized likelihood function [1]. In order to compute the likelihood of a given reward function during sampling, it is required to compute the Q-values for all the state-action pairs in the demonstration set, which means solving a reinforcement learning (RL) problem given the Markov Decision Process (MDP). Therefore, Bayesian IRL is indeed a very computationally expensive algorithm.

In order to make our human experiment environment tractable by Bayesian IRL, the virtual room is discretized into a 2D gridworld of size $32\times$ 24 with 0.2×0.2 m^2 cells. Each cell is a state in the MDP. The actions are discretized into 8 directions so that an agent can move to any adjacent state in the gridworld. The (center) location of targets, obstacles and waypoints are treated as different feature points, which contribute to each state's feature by distance. The problem is formulated as learning the weights for the three different features: targets, obstacles and waypoints. The three features are represented using three different continuous values at each state. More specifically, the closer a state is to an target/obstacle/waypoint, the higher the feature value for the particular object at that state. The reward at any given state is computed as the linear combination of these features using their corresponding weights. The observations are a set of state-action pairs extracted from the human's trajectory, which are fitted to the discretization of the space.

The parameters for Bayesian IRL are set empirically. The confidence factor α is set at 80 and the chain length is set to be 3000 (since there are only three values, i.e. feature weights, to be tweaked, which is relatively small). A value of 0.5 is used as the discount factor for MDPs with the assumption that the decision making process of humans tends to prefer immediate rewards.

References

1. Ramachandran D, Amir E. Bayesian inverse reinforcement learning. In: Proceedings of the 20th International Joint Conference on Artificial Intelligence. Morgan Kaufmann Publishers Inc.; 2007. p. 2586–2591.
